# Current Status and Prospects of Research on Sensor Fault Diagnosis of Agricultural Internet of Things

**DOI:** 10.3390/s23052528

**Published:** 2023-02-24

**Authors:** Xiuguo Zou, Wenchao Liu, Zhiqiang Huo, Sunyuan Wang, Zhilong Chen, Chengrui Xin, Yungang Bai, Zhenyu Liang, Yan Gong, Yan Qian, Lei Shu

**Affiliations:** 1 College of Artificial Intelligence, Nanjing Agricultural University, Nanjing 210031, China; 2 School of Population Health Sciences, King’s College London, London WC2R 2LS, UK; 3 College of Engineering, Nanjing Agricultural University, Nanjing 210031, China; 4 College of Engineering, Northeastern University, Boston, MA 02115, USA; 5 School of Engineering, University of Lincoln, Lincoln LN6 7TS, UK

**Keywords:** agricultural Internet of Things, sensors, fault diagnosis, deep learning

## Abstract

Sensors have been used in various agricultural production scenarios due to significant advances in the Agricultural Internet of Things (Ag-IoT), leading to smart agriculture. Intelligent control or monitoring systems rely heavily on trustworthy sensor systems. Nonetheless, sensor failures are likely due to various factors, including key equipment malfunction or human error. A faulty sensor can produce corrupted measurements, resulting in incorrect decisions. Early detection of potential faults is crucial, and fault diagnosis techniques have been proposed. The purpose of sensor fault diagnosis is to detect faulty data in the sensor and recover or isolate the faulty sensors so that the sensor can finally provide correct data to the user. Current fault diagnosis technologies are based mainly on statistical models, artificial intelligence, deep learning, etc. The further development of fault diagnosis technology is also conducive to reducing the loss caused by sensor failures.

## 1. Introduction

The Internet of Things (IoT) is widely used in various fields, such as intelligent healthcare [1], smart agriculture [2], smart transportation [3], the smart home [4], and the smart city [5,6]. The agricultural Internet of Things (Ag-IoT) has been widely used in agricultural scenarios such as field planting, livestock and poultry breeding, aquaculture, facility horticulture, and agricultural product logistics traceability as an important development direction of “Internet +” agriculture to realize the comprehensive perception of the agricultural production process, intelligent decision analysis, and early warning, and finally achieve the goal of precision agriculture and intelligent agriculture. The Ag-IoT has been used for a long time in many countries, and the technology is relatively mature. The United States began to use computers for intelligent irrigation and production management in the 1980s. Ag-IoT coverage on large farms in the United States has reached 80%. In 2004, Japan’s Ministry of Internal Affairs and Communications proposed the U-Japan plan, which included Ag-IoT technology developed primarily by NEC, Fujitsu, Hitachi, and Mitsui. China proposed the “Sensing China” strategy in 2009 [7], and related research and the application of Ag-IoT also began to develop rapidly.

Sensors, the core of Ag-IoT, are primarily used to collect various data in the agricultural production process. Furthermore, sensors can be integrated with other systems to enhance automatic control capabilities. However, agricultural sensors are prone to frequent faults due to poor deployment environments and remote deployment locations. For example, when a temperature and humidity sensor is biased or drifts, the irrigation system will not work correctly [8]. If the sensor in the robot system fails, it may face paralysis [9,10]. Incorrect detection values of sensors will lead to wrong decisions in intelligent agriculture systems [11,12]. Unmanned aircraft may lose control or even crash due to problems with the gyroscope and accelerometer [13]. Due to the influence of the external environment, sensor aging is also a source of sensor failures, leading to incorrect decisions [14] and failure to transmit information normally [15]. In different application scenarios, sensor failures can cause significant human, economic, and environmental losses and reduce the quality of IoT service.

**Figure 1:**
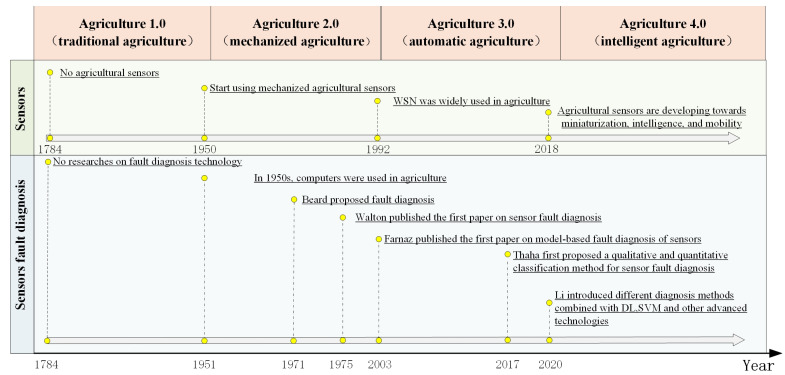
Timeline of development in sensor fault diagnosis technology in Ag-IoT systems.

Since Beard [16] proposed fault diagnosis technology in 1971, experts and scholars have conducted extensive research in sensor fault diagnosis. Figure 1 depicts the research progress of sensor fault diagnosis technology [16,17,18,19,20,21,22,23,24,25,26]. Early sensor fault diagnosis methods relied on experience and simple ways to pinpoint the location and cause of a problem, such as sensor redundancy, function redundancy, and characteristics tracking [27]. Sensor redundancy is the use of multiple sensors to measure the same parameter, the linear combination of the measured values of several sensors is converted into an estimated value, and the final comparison of the measured value with the estimated value is used to determine whether faults occur. This method, however, raises hardware costs. Functional redundancy uses information from each sensor in the system to determine whether there is a fault via the relationship between sensors in heterogeneous and homogeneous locations. Characterization tracking determines whether the sensor values are within acceptable limits.

With the widespread adoption of Ag-IoT and the rise of machine learning, a new method for sensor fault diagnosis has emerged. A fault diagnosis model that has been trained with a large amount of data has a faster and more accurate effect [28]. In particular, as deep learning exhibits powerful data representation learning and analysis capabilities, it can meet the requirements of high-order, nonlinear, adaptive feature extraction for sensor fault diagnosis [29]. A sensor fault diagnosis method based on Ag-IoT can overcome the limitations of early sensor fault diagnosis by fully using more technical support, such as machine learning, statistical analysis, and signal processing, to realize remote real-time online fault diagnosis, even without the presence of maintenance personnel. The system can diagnose itself and recover or isolate the corresponding fault, allowing the sensor network to continue operating normally after the fault occurs while improving the intelligent level of fault diagnosis [30].

This paper offers an overview of Ag-IoT sensor fault diagnosis technology. First, sensor networks and sensors are introduced, and the current state of sensor research is reviewed. The sensor fault is investigated using Ag-IoT characteristics. The common fault root causes, fault types, and sensor fault characteristics are discussed. The methods and strategies for detecting sensor faults are thoroughly examined. Finally, the future direction of sensor fault diagnosis development is discussed.

## 2. Background of Sensors and Sensor Networks in the Ag-IoT

### 2.1. Sensors and Sensor Networks

Smart agriculture requires the deployment of various sensors on farmland, crops, animals, and agricultural equipment. High-quality, high-resolution, high-reliability professional agricultural sensors must be designed and developed to sense the physiological markers of agricultural production environments, animals, and plants. According to the different detection objects, sensors can be divided into many types, such as gas sensors, temperature and humidity sensors, illuminance sensors, nutrient element sensors, and so on.

Sensor networks are broadly classified into two types: wired sensor networks and wireless sensor networks. Wired sensor networks do not meet the requirements of remote deployment areas, wide monitoring areas, and low cost due to the shortcomings of complex wiring and the high cost of wired sensors. In comparison, wireless sensor networks have the advantages of flexible installation and low cost, and they are being used in an increasing number of agricultural fields. The self-organization of a large number of wireless sensors forms a Wireless Sensor Network (WSN). A WSN can remotely transmit data from various detection objects in the monitoring area to terminal users in real time and quickly. It can be deployed in remote areas to overcome wired limitations. A WSN consists of sensor modules, processor modules, wireless communication modules, and energy supply modules. The sensor module collects environmental information and converts the collected signal to digital analog; the processor module filters and processes the data; the wireless communication module sends the collected information to the sink node or base station; and the energy supply module is in charge of the entire wireless sensor’s energy supply, including power supply, solar power, and wind power. Wireless sensors are typically dispersed at random throughout the monitoring area. The collected data are transmitted multi-hop between nodes before being wirelessly transmitted to the base station [31]. With the advances in communication technology, many different types of wireless networks have emerged, as shown in Table 1. The deployment nodes of agricultural sensors can be adjusted to meet the various service requirements of agricultural applications.

### 2.2. Agricultural Internet of Things

**Figure 2:**
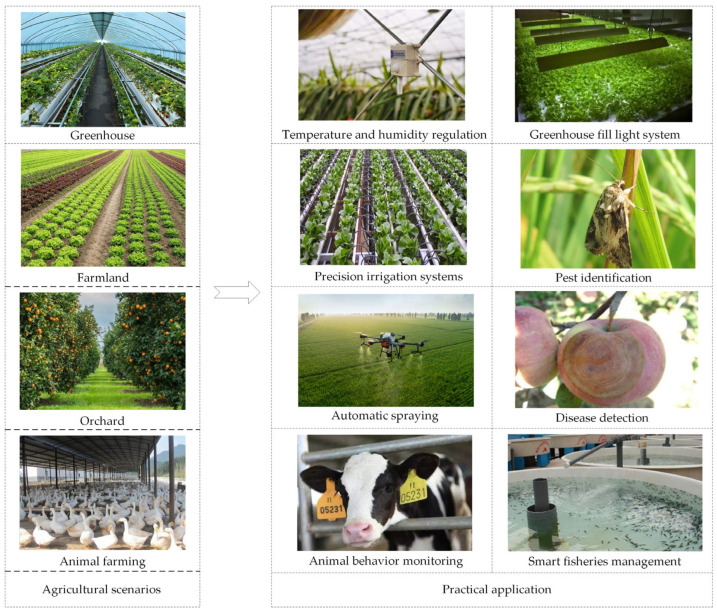
Practical applications of IoT in agriculture.

The world recognizes the IoT as the third wave of the world’s information industry after computers, the Internet, and mobile communication networks. It can realize a comprehensive network of people and people, people and things, and things and things. As agriculture enters the 4.0 era, the current planting and breeding industry are showing the characteristics of scale and refinement through the integration of agriculture with emerging technologies such as the IoT, big data, artificial intelligence, etc., via various sensors to collect information, such as temperature, humidity, nitrate content in the soil, conductivity, and PH [37]. Monitoring and controlling essential factors that affect crop growth and yield can significantly reduce economic losses through the timely discovery of risks in the breeding process. The continuous emergence of intelligent agricultural equipment will promote the automation and intellectualization of agriculture. As shown in Figure 2, IoT technology is currently widely used in agriculture, including precision agriculture, livestock monitoring, smart greenhouses, fisheries management, and weather tracking [38,39].

### 2.3. Research Status of Ag-IoT Sensors

The implementation of Ag-IoT systems is reliant on multi-functional intelligent sensors. The wide range of sensors includes agricultural environmental sensors, agricultural animal and plant life information sensors, agricultural product information sensors, and other agricultural sensors (such as position information and pressure information sensors). The primary function of agricultural sensors is to collect data on numerous agricultural factors, including temperature, humidity, light intensity, and gas concentration in the field planting industry; ammonia and carbon dioxide levels, air quality, temperature, and humidity in the livestock and poultry breeding industry; dissolved oxygen, salinity, carbon dioxide, and ammonium nitrogen in aquaculture; and temperature, humidity, light intensity, and carbon dioxide in facility horticulture. In terms of principle and method, agricultural sensors based on an electrical sensing mechanism are primarily used for temperature and humidity sensing [40]. Sensors utilizing a photoelectric sensing mechanism are mainly employed to measure light intensity, gas composition, and phenotypic object displacement. Sensors based on an electrochemical sensing mechanism are primarily used to detect temperature, humidity, and gas concentrations, including oxygen, carbon dioxide, and ammonia. However, at present, the intelligence and technological maturity of agricultural environment sensors are low. Although numerous companies are conducting this type of sensor research, only a few companies research animal and plant physiological information sensors due to technological and financial limitations. Table 2 displays representative agricultural sensor systems in the Ag-IoT.

## 3. Types and Characteristics of Sensor Faults

Sensors in the Ag-IoT structure are prone to many faults during data acquisition and transmission. A sensor fault refers to a sensor whose sensing or transmitting data is significantly different from other sensor data; the data don’t conform to the expected normal behavior or are highly consistent with the defined characteristics of fault data [63]. According to the acquisition process analysis, agricultural sensors are typically deployed in unattended and harsh indoor or outdoor environments. Wireless sensors usually have limited resources such as power, memory, and computing resources. Long-term use damages the sensor module, power module, and other wireless sensor hardware, resulting in irreparable faults that prevent the sensor from collecting data normally.

According to the transmission process analysis, the complex agricultural environment, such as crops, terrain, and greenhouse walls, impacts wireless sensor communication. Due to a fault in the agricultural wireless sensor communication module, external attacks, and limited communication capabilities, data transmission fails, and sensor data collected cannot normally be sent to the server [64].

### 3.1. Types of Sensor Faults

**Figure 3:**
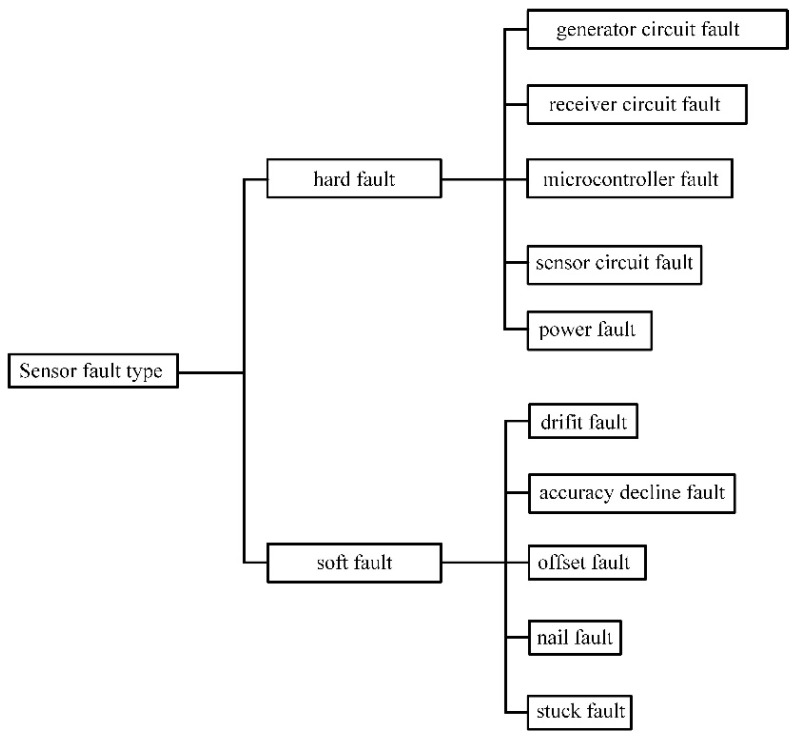
Types of sensor faults in agricultural sensor systems.

The hard fault and soft fault types are distinguished by the duration of the sensor fault. Hard faults are permanent faults that persist until the fault recovery phase. Soft faults are temporary faults that disappear after a certain period. Figure 3 depicts the classification of hard faults and soft faults.

#### 3.1.1. Hard Fault

A hard fault refers to the damage or failure of the agricultural sensor’s hardware, resulting in a sensor fault. A hard fault prevents the sensor from collecting and transmitting data normally, including a transmitter circuit fault, receiver circuit fault, microcontroller fault, sensor circuit fault, and power fault. A sensor with poor circuit contact is highly susceptible to short-circuit faults. Circuit disconnection results in an open circuit sensor fault. The poor working environment causes sensor hardware to malfunction. Most of these faults are permanent and require the replacement of defective hardware or maintenance circuits.

Researchers report that power supply failures are the primary cause of sensor data errors [65]. The primary factor limiting wireless sensors is the power supply [66]. Wireless sensors must operate for years or even decades after deployment, particularly when some nodes are deployed in remote locations, making it difficult to regularly meet the power maintenance requirements. A power failure causes the sensor to be unable to collect information correctly, or transmit data normally, and may even cause the failure of the entire agricultural sensor network. The energy consumption of wireless sensors is primarily attributable to monitoring, data transmission, and data reception. In the paper [67], the impact of power faults on WSNs is categorized into four groups: node, link, routing path, and global fault. A node fault may occur when the power supply falls below the node’s operating level. When a node’s power supply is insufficient, its communication range is diminished, resulting in a link fault. When one or more nodes have link faults, the WSN becomes less efficient, resulting in routing path faults. When node or link faults occur in key nodes, WSN communication is interrupted, resulting in a global fault.

#### 3.1.2. Soft Fault

According to the fault scheme, sensor soft faults can be divided into drift faults, bias faults, accuracy decline faults, stuck faults, and spike faults [68,69].

(1) Drift fault

A drift fault refers to the measured value and the real value of the sensor changing with time. At this time, the output value of the sensor increases at a constant rate. Such faults must be diagnosed and restored in time, otherwise, significant measurement errors occur [47]. In addition, drift faults cause severe damage to chemical sensors and biosensors if they are not readily detected at an early stage and need to be diagnosed early [70].

(2) Bias fault

A bias fault (offset fault) refers to a constant value added to the sensor measurement value, and the sensor output result deviates from the normal value [71]. When the manipulator sensor has a bias fault, it leads to poor regulation or tracking performance and even affects the stability of the control system. In addition, faulty data may lead to wrong decisions, making unnecessary component replacement or task termination in the system [72].

(3) Stuck fault

A stuck fault is a sudden sensor measurement error with a constant measurement value. This flaw may vanish over time, but it will persist for a considerable time. The fault characteristics are evident and simple to identify [25]. Sometimes, the causes of a stuck fault are identical to those of other faults. For instance, a clogged pressure sensor causes a stuck fault, while a blocked flow sensor causes a decline in accuracy [73].

(4) Accuracy decline fault

An accuracy decline fault indicates that the average value of the sensor measurement does not change, but the variance of the output value increases, resulting in a decline in measurement accuracy [74]. This fault frequently occurs in sensors, and early detection is crucial for monitoring. However, current research focuses primarily on drift faults, bias faults, and stuck faults, while accuracy decline fault research is scarce [75].

(5) Spike fault

A spike fault refers to a large amplitude spike in the measured value of the sensor, which often occurs in sensors. One of the reasons is the loose connection within the sensor node [76,77]. When a fault occurs in sensors, the system makes wrong decisions, such as spike faults in livestock and poultry houses that exceed the set environmental threshold, causing fans, heaters, and other equipment to be turned on or off.

### 3.2. Characteristics of Sensor Faults

(1) High spatial-temporal correlation

The data collected by the sensor are a time series consisting of an ordered collection of measurement values collected at regular time intervals. Consequently, the influence of historical and future data should be considered during fault diagnosis. Moreover, since a large number of sensors of the same type are typically deployed in a particular area, there is a high spatial correlation between sensors, so other sensors should be consulted during the fault diagnosis process.

(2) Frequent abnormal data

Fault data include bias fault, drift fault, and so on, mainly caused by sensor faults or damage. The difference between abnormal data and fault data is small, but the duration is usually short. Because agricultural sensors are typically deployed in outdoor harsh environments, sudden changes in the environment can result in random changes, measurement, and recording errors, and thus make sensors produce outliers.

(3) Different fault duration

Due to extreme weather fluctuations or the influence of other organisms, the sensor may experience a brief period of faults. When such an error occurs, the hardware and software of the sensor are normal, and the error is quickly rectified. Due to the deterioration of sensor hardware and software or insufficient power, fault duration is lengthy, and most faults are permanent. Such faults cannot recover independently; fault diagnosis and recovery are required.

## 4. Strategies for Sensor Fault Diagnosis

Based on the different fault diagnosis methods performed in the Ag-IoT system structure, fault diagnosis is divided into centralized and distributed methods.

### 4.1. Centralized Strategy

The most common solution in agricultural sensor fault diagnosis is a centralized approach. The central node or base station diagnoses each sensor node’s condition. The central node’s limitless resources (such as batteries and computing power) lengthen the lifespan of agricultural WSNs [78]. A trained fault diagnosis model is stored in the central node that periodically sends requests to the network to detect the state of the entire sensor network and diagnoses and locates faulty sensors after analyzing agricultural sensor data. Lau et al. [65] proposed a centralized fault detection method for WSNs based on the Naive Bayes framework to detect sensor battery issues; the diagnosis process was not performed in each sensor node, thereby reducing battery load. Salah et al. [79] proposed a centralized policy sensor fault scheme based on SVM that performed fault diagnosis at the cluster head and utilized fewer sensor resources.

This method has minimal hardware requirements for agricultural sensor nodes, requires no additional computing and memory resources, and extends the sensor’s service life. However, a large amount of information is sent to the central node, which causes network congestion and slows down detection speed; due to fault diagnosis at the central node, it is difficult to meet real-time requirements; in the process of data transmission to the central node or base station via multiple hops, the pressure on the sink node increases, consuming the sink node’s resources.

### 4.2. Distributed Strategy

Due to the small number of base stations in agricultural scenarios, weak signals, and low network coverage, agricultural sensor data cannot be transmitted to the central node or base station in some areas, making it challenging to implement centralized fault diagnosis. In addition, the centralized fault diagnosis strategy increases network traffic, resulting in network congestion and an inability to meet real-time fault diagnosis requirements. The distributed fault diagnosis strategy effectively resolves this issue. Distributed fault diagnosis is located between sensor nodes, eliminating the need to send agricultural sensor data to a central node or base station for diagnosis and satisfying real-time specifications. Sana et al. [80] proposed a distributed sensor fault diagnosis system based on a machine learning algorithm, which implemented fault detection in the sensor and performed the fault diagnosis on the central node, thereby ensuring the real-time performance of the fault diagnosis and reducing the number of calculations performed on the sensor.

The greater the number of decisions the sensor node makes, the less information is transmitted to the central node, thereby accelerating the detection rate. However, the hardware requirements of the sensor node are high, and the node for fault diagnosis requires specific memory, computing, and battery resources. The memory resources of wireless sensor nodes are on the order of kilobytes, and the operation speed is on the order of MHz. Conversely, due to the limited resources carried by the sensor nodes, the fault diagnosis method must meet the requirements of being lightweight and having high performance, allowing for fast and accurate diagnosis despite limited computing and storage resources without excessive battery consumption. Data-driven fault detection methods require fewer resources than model-driven ones [81]. Some researchers [82] investigated using lightweight fault diagnosis algorithms to reduce sensor resource consumption during the fault diagnosis procedure.

## 5. Intelligent Fault Diagnosis of Sensor Faults

**Figure 4:**
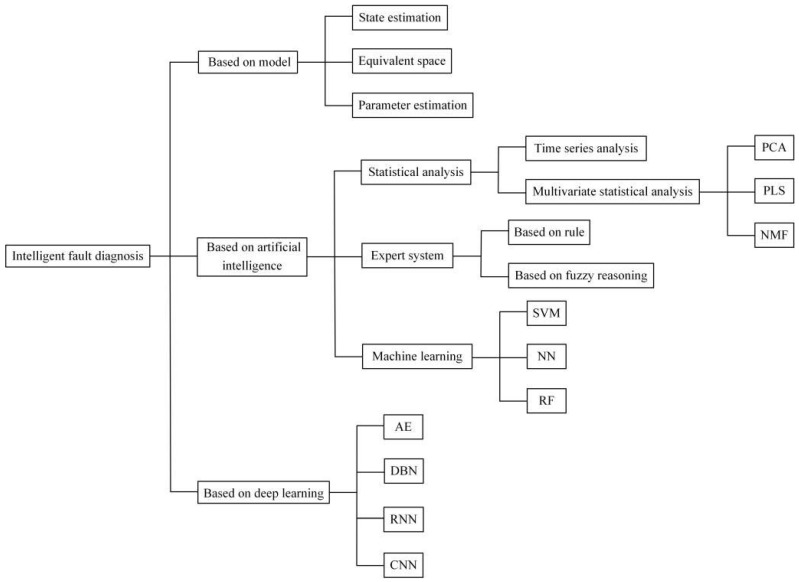
Classification of intelligent fault diagnosis methods.

The purpose of sensor fault diagnosis is to detect the fault data in the sensor, and restore or isolate the faulty sensor so that the sensor can finally provide normal data to the user. Traditional fault diagnosis techniques mainly rely on manual judgment, and the experience and expertise of engineers determine the accuracy of judgment. However, with advances in science and technology, agriculture has entered the 4.0 era, and the data collected by sensors are multi-dimensional and large-scale, and the monitoring objects often have coupling relationships and affect each other. Relying only on manual machine fault diagnosis struggles to meet the current needs in terms of accuracy and real-time. In factory farming scenarios, users want an automated way to reduce labor costs and improve diagnostic accuracy. The introduction of intelligent fault diagnosis (IFD) technology is expected to achieve this goal. IFD refers to the application of machine learning theories (such as artificial neural networks (ANN), support vector machines (SVMs), and deep neural networks (DNNs)) in machine fault diagnosis [83], adaptively learning machine fault diagnosis knowledge from the collected data, automatically establishing a relationship model between the collected data and the health state of the machine, and realizing the automation and intelligence of sensor fault diagnosis technology. This section divides the sensor fault diagnosis technology into three methods: model-based, artificial intelligence-based, and deep learning diagnosis-based, as shown in Figure 4. Relevant sensor fault diagnosis approaches are shown in Table 3.

### 5.1. Model-Based Fault Diagnosis Methods

The model-based fault diagnosis method was proposed by Beard of the Massachusetts Institute of Technology in 1971 [16] and refers to using knowledge of the system’s structure, behavior, and function to diagnose the fault of the system [84]. Based on the model, fault diagnosis methods are divided into three types: state estimation, equivalent space, and parameter estimation [85]. The approach consists of two main steps: residual error generation and residual error evaluation. This diagnosis method requires understanding the mechanism of the research object and establishing a mathematical model, using the system’s structure, behavior, or function to simulate the model, and then making a decision based on the difference between the actual measured signal and the model measured signal. By generating residual signals to achieve fault diagnosis [86], the occurrence of a fault can be detected by evaluating whether the resulting residual exceeds a threshold. Vasso et al. [87] designed a model-based distributed fault diagnosis framework for detecting and isolating multi-sensor faults in heating, ventilation, and air conditioning systems. The adaptive threshold framework ensures the proposed method’s robustness against modeling uncertainties and measurement noise. Zhang et al. [88] proved that a Principal Component Analysis (PCA)-based sensor fault detection method is less efficient in fault detection under minor deviation fault conditions. Yu et al. [89] proposed a model-based fault diagnosis scheme that uses open-circuit voltage residuals and capacity residuals to deduce fault values of voltage and current sensors for battery fault diagnosis. Yan et al. [90] proposed a Cp_PLV model combining an unsupervised learning method with change point recognition. Experiments show that the predictive effect of the model is good and can fault diagnose the sensor in complex working environments.

Due to the fact that the model-based fault diagnosis method employs fundamental knowledge for fault diagnosis, it has improved fault interpretability. It can also meet the real-time requirements of agricultural sensor fault diagnosis. In practice, however, the model is uncertain, and it is difficult to establish a model that corresponds to other agricultural sensors. It is challenging to ensure fault diagnosis accuracy with an unmatched model, which cannot detect the fault value and is not universal.

### 5.2. Artificial Intelligence-Based Fault Diagnosis Methods

With the widespread adoption of Ag-IoT, the uncertainty and complexity of the sensor system have gradually grown, as has the difficulty of developing an accurate mathematical model. The artificial intelligence-based fault diagnosis method treats the system as a black box, does not need to understand the structure and principle of the system and the precise mathematical model of the diagnostic object, and uses a large amount of sensor system data for fault diagnosis [91]. The method relies primarily on real-time or historical data and comprises artificial intelligence algorithms and statistical data processing. The primary benefit of this method is that it does not require a precise system model, and it has been utilized successfully for agricultural sensor fault diagnosis. Common fault detection techniques based on artificial intelligence include statistical analysis, expert systems, and machine learning.

#### 5.2.1. Statistical Analysis Methods

The statistical analysis method primarily involves a sensor’s randomly distributed data collection. The sensor is deemed defective when the probability of the data instance generated by the model is extremely low. The primary components of this method are time series analysis and multivariate statistical analysis.

The time series analysis method examines the characteristics of sensor data from a time series perspective, estimates the predicted value of the sensor data, and determines whether the sensor is faulty by comparing the difference between the predicted value and the actual value. Hao et al. [92] designed a module based on time series theory to detect sensor faults in the navigation system of autonomous mobile agricultural robots. Simple and effective, the time series method was able to explain the relevant results intuitively. However, agricultural sensors are typically deployed outside, where the environment constantly changes, causing the sensor data to fluctuate greatly and be prone to error. Due to the difficulty of a single sensor explaining the complexity of agricultural environments, multivariate statistical analysis methods that combine data from multiple sensors for fault diagnosis are crucial. Nonnegative Matrix Factorization (NMF) [14], Partial Least Squares Regression (PLS) [93], and PCA [94] are the three multivariate statistical analysis methods.

After establishing the probability statistics model, the model can effectively detect sensor system faults. The model is highly interpretable; however, when using time correlation to detect faulty data, a sudden change in the data distribution will reduce the time correlation and make sensor fault diagnosis more difficult. The statistical analysis method has the following drawbacks: the nonparametric statistical model struggles to meet real-time requirements; the parametric statistical model is ineffective in practical applications due to a lack of data distribution knowledge in the sensor system; the histogram does not take into account the relationship between multivariate data and only applies to univariate data.

#### 5.2.2. Expert System Methods

The method based on an expert system is used to diagnose faults based on the experience of experts and maintenance personnel involved in producing agricultural sensors. It is primarily divided into rule-based expert systems and expert systems with fuzzy reasoning. An expert system is a structured knowledge system that imitates human experts to solve problems in a particular domain. Expert system components include a knowledge base, a rule base, an inference engine, a human-computer interface, and an explanation facility [95]. The knowledge base is the key to ensuring the expert system’s accuracy, which comprises the operators’ theoretical and professional knowledge. The system operates as follows: the data to be diagnosed is input through a human-computer interface, the inference engine matches the current known conditions, information, and rules of the knowledge base, and the user is then presented with the conclusion of the matching rules [96,97]. Prasenjit [98] proposed a WSN fault node classification and management scheme based on fuzzy rules, which detected the sensor fault state and divided it into various categories to guarantee the reusability of fault nodes. Pooja et al. [99] proposed a hardware fault diagnosis model for sensor nodes based on a fuzzy inference system with three inputs, developed 27 fuzzy rules based on the status of the transmitter, receiver, and battery, and categorized the nodes as normal nodes, end nodes, and dead nodes. This scheme’s detection accuracy and misdiagnosis rate have been enhanced.

A mathematical model is not required by the expert system-based technique for determining what is wrong. It employs the rules and information we already possess to determine what is wrong with an agricultural sensor system. It has outstanding advantages in the application of nonlinear systems because it has good performance and strong learning ability in dealing with known faults. Nevertheless, there are still things that could be improved with this method: As more and more types of sensors are utilized, the complexity of the sensor system must increase. This makes it difficult to determine why the sensors are malfunctioning. The current expert systems for fault diagnosis lack universality and adaptability, and each system operates independently. This is extremely wasteful. Users are interested in the location and timing of agricultural sensor failures.

#### 5.2.3. Machine Learning Methods

There is no simple correspondence between fault types and fault characteristics due to the complexity and unpredictability of sensor systems. The fault diagnosis method based on machine learning can train support vector machine (SVM), artificial neural network (NN), and other machine learning algorithms using normal data and fault data, and then diagnose the fault of sensors.

SVM is a machine learning algorithm that solves binary classification problems [100]; sensor fault diagnosis is based on small samples. Consequently, the SVM model is ideally suited for sensor fault diagnosis and has been widely implemented in the field [101,102]. Yang et al. [103] divided multiple fault combinations according to the correlation of faults. They developed multiple SVM models based on these fault combinations to combat the misdiagnosis caused by insufficient and unreliable training data in traditional SVM models. Each SVM model assigned positive and negative labels to fault samples, corrected various label combinations using DS theory, and then determined the fault type. Although the fault identification accuracy of this scheme has been enhanced, the DS-theory-based correction rate must be improved. Deng et al. [104] proposed OS-LSSVM for sensor fault detection, which solved the problem of LS-SVM lacking sparsity, enabled online fault diagnosis, and increased calculation speed. The SVM method primarily solves the quadratic programming problem; however, the calculation method based on quadratic programming restricts real-time performance and is unsuitable for online fault diagnosis. Han et al. [105] proposed an LS-SVM model that reduced the computational complexity by transforming the quadratic programming problem into a linear equation. The model’s execution time was reduced by 36.7% compared to the SVM model, and the diagnostic accuracy was also enhanced. In addition, Liu et al. [106] proposed a KNN-FSVM fault diagnosis scheme, which trained SVM by only selecting boundary data, thereby reducing the need for computing and storage resources. This was a solution to the problem that the computing time of SVM increases exponentially with the amount of data, as do the requirements for computing and storage resources.

While traditional SVM models can diagnose systems with a small number of data samples, as sensor performance becomes more complex, the types of sensor faults increase, and the accuracy of the classification model decreases. Therefore, the SVM-based fault diagnosis method is not suitable for use alone in the current diversified agricultural production mode.

ANN is a mathematical model that simulates the mechanism by which the nervous system of the human brain processes complex information based on the fundamental principles of neural networks in biology [107]. ANN is a nonlinear model primarily composed of neurons that are incorporated into three layers, referred to as the input layer, hidden layer, and output layer, with the ability to simulate any continuous nonlinear function and learn from samples, expressing the learned fault diagnosis knowledge with neural network connection weights, which has been utilized in the fault diagnosis of complex systems [108,109]. Back-propagation Neural Network (BPNN) [110] is currently the most widely used neural network model. Researchers created it to address the challenges of multi-layer neural networks. Hu et al. [111] employed BPNN for temperature sensor fault diagnosis. The model selected the most recent local data, reducing the computation required and enabling online detection. In order to eliminate data noise and improve the accuracy of neural network fault diagnosis, Shi et al. [112] incorporated wavelet denoising technology into the BPNN, which enhanced the BPNN’s fault detection rate. Guo et al. [113] proposed an EFMSAE-LSTM method for predicting the time series of mechanical failures, which can accurately predict the failure time series of most key mechanical components. Mariam et al. [114] performed fault diagnosis for HVAC sensors using a self-associative neural network and compared this technique to principal component analysis. Not only was the diagnostic accuracy of this method significantly enhanced, but it could also diagnose multiple sensors simultaneously. Precision agriculture is characterized by massive normal agricultural data and small sample fault data. The extreme imbalance of these data renders the neural network’s fault diagnosis method less stable; The parameters of the model need to be adjusted repeatedly to meet the requirements of the practical application, which requires good model training experience.

Although machine learning-based fault diagnosis methods have high time and money costs in network training, they also require relatively large training data samples. However, considering the practical application of sensors in agriculture, the data collected is susceptible to many factors, such as weather, region, season, and changes in the growth stage of animals and plants. Especially for the current large-scale and industrialized farming pattern, it is difficult or even impossible to obtain accurate system models. Small changes in the system can lead to poor failure detection response. Therefore, compared with the fault diagnosis methods based on models and expert systems, the fault diagnosis methods based on machine learning have good generalization ability and wider applicability, which are suitable for application in the era of diversified and integrated agriculture 4.0.

### 5.3. Deep-Learning-Based Fault Diagnosis Method

With the advancement of hardware and algorithms and the exponential growth of data, fault diagnosis has shifted from machine learning to deep learning [115]. Deep learning, a subfield of artificial intelligence, is the newest data analysis and image processing technology. Compared with traditional statistical learning models, deep learning models have a stronger ability to extract underlying information from data. The structure of deep learning models is usually formed by multi-layer neural networks, which can be flexibly changed to meet different practical needs. In addition, multiple layers of data processing units are assembled to form a deep architecture. It has powerful learning capabilities and can extract features automatically from input data. Hierarchical features are represented by low-level features, which can efficiently and quickly solve complex problems and have good portability, making them suitable for sensor fault diagnosis. Autoencoders (AEs), Deep Belief Networks (DBNs), Recurrent Neural Networks (RNNs), and Convolutional Neural Networks (CNNs) are the current deep learning models for sensor fault diagnosis.

#### 5.3.1. Autoencoder (AE)

An AE is an unsupervised learning neural network that uses the back-propagation algorithm to make the output value equal to the input value [116], which can effectively extract low-dimensional data features. Encoding and decoding are the two components of an AE. The encoder compresses the input into a representation of latent space, and the decoder reconstructs the information from the latent space representation. With the advent of deep learning, researchers have created additional AE model types. For instance, to extract abstract features from data, several AEs are stacked to form a Stacked Autoencoder (SAE), and a Denoising Autoencoder (DAE) was developed to improve the anti-noise capability of neural networks [117].

Under the premise of training on small data samples, AE can achieve efficient fault diagnosis when combined with other classification methods [118]. The use of AE for fault diagnosis has received considerable attention in recent years. Luo et al. [119] designed an AE-distributed fault diagnosis system with only three layers of the network, which was used for fault diagnosis in sensors, and the model was trained in the cloud to address the issue that deep learning methods consume a great deal of computing and communication resources. Jia et al. [120] proposed a normalized sparse AE model for intelligent fault diagnosis, learning various meaningful features from the input signal. The translation in-variant features were obtained in the feature layer, and then the fault was identified in the output layer, and the accuracy rate of the model test reached 99.92 percent. In addition, due to the model training phase, AE automatically extracts non-fault-related features, which hinders fault diagnosis performance. Wang et al. [121] proposed a supervised SAE model capable of extracting fault-related deep features and configuring fine-tuned network initial parameters. This method improved classification precision with fewer iterations. The local minimum problem exists in the traditional AE method for fault diagnosis. Through batch regression, hyperparameter optimization, and other techniques, the model can be made to closely resemble the actual working conditions of the sensor, which is advantageous for resolving this issue. Mallak et al. [122] propose a two-stage fault diagnosis method that uses an LSTM autoencoder to perform a separate fault detection stage in advance. This method can effectively catch rare faults.

AE has the advantages of strong learning ability, simple structure, and easy training. However, traditional AE only uses a single-layer encoder for feature extraction, which struggles to extract deep features and has limited data processing capabilities. As the modern agricultural production process gradually presents the characteristics of modularization and specialization, it is suitable for application in distributed fault diagnosis and processing small samples of univariate data.

#### 5.3.2. Deep Belief Network (DBN)

A DBN is a probabilistic generative model of multiple Restricted Boltzmann Machines (RBM). Each RBM consists of only two layers of neurons, the visible layer and the hidden layer, which are connected by a symmetrical weighted connection matrix [123,124]. The visible layer inputs training data, whereas the hidden layer extracts features. In each RBM, the data vector is used to infer the hidden layer, which is then used as the data vector for the subsequent layer to improve the probability variation lower bound of the training data. As feature extraction is a crucial step in the diagnosis process, DBN employs an unsupervised greedy layer-by-layer method to obtain high-level feature representation, which can effectively eliminate the complexity and uncertainty introduced by manual feature extraction and enhance the intelligence of fault diagnosis [125,126]. In addition, DBN has the capacity to manage high-dimensional and nonlinear data, thereby resolving the issues of data dimensional disaster and inadequate diagnostic capability.

Since methods like SVM and ANN can only detect faulty data but not faulty sensors [127], Mandal et al. [128] proposed a method based on DBN and the generalized likelihood ratio test. DBN was utilized to classify fault data and normal data, the faulty sensor was identified by the maximum deviation between the fault data and the average value of the normal data, and the quantity of fault data and the generalized likelihood ratio test determined the fault mode. However, this method only detected faults when the signal exceeded the threshold; therefore, other methods are required to detect faults when the signal was below the threshold. Due to the loss of potentially valuable information in the original data caused by the layer-by-layer feature compression of the DBN model, Wang et al. [129] employed the strategy of repeatedly stacking the original data during the training phase in order to fully extract the valuable information by extending the DBN model. Each RBM was trained with the original data so that the extracted features were highly correlated with the original data, and potentially valuable information was preserved. Compared to the conventional DBN model, the accuracy rate has been enhanced.

DBNs can extract advanced features from large amounts of data and directly use raw signals to build end-to-end intelligent diagnostic models to reduce reliance on expert experience and known knowledge. They can fuse the time domain and frequency domain characteristics of each sensor signal and apply this to DBN training, which can not only realize fault diagnosis but also diagnose fault types. They are suitable for applications in advanced composite sensor systems.

#### 5.3.3. Recurrent Neural Network (RNN)

An RNN is a type of neural network that processes sequence data. Its most distinguishing characteristic is that the output of neurons is transmitted to the input of itself or other neurons. Its properties can connect the nodes between hidden layers, and the input of the hidden layer incorporates the input of the input layer and the output of the hidden layer at the time of its creation. This concatenated neural network structure is appropriate for time series data and can preserve data dependencies [130]. Later, Hochreiter et al. [131] proposed a Long Short-Term Memory (LSTM) to improve the traditional RNN model to address the issue that RNNs are not good at long-term memory.

**Table 3:** Development and application of intelligent sensor fault diagnosis approach.

	Diagnostic Method	Diagnostic Strategy	Application and Improvement	Applicable Sensor	Applicable Fault Type	References
Model-Based	-	Distributed	Adaptive thresholds to ensure robustness to noise and modeling uncertainty	Temperature, CO_2_	-	Zidi S et al. (2015) [79]
	-	-	Verification of the reliability of PCA for fault diagnosis of air conditioner sensors	Temperature	Soft fault	Jan et al. (2017) [80]
	State estimation	-	Fault diagnosis of battery sensor based on PLS and UKF	Voltage, current	-	Banerjee et al. (2021) [81]
Artificial Intelligence-Based	TSA	Centralized	Detection of sensor faults of agricultural robot navigation system based on time series theory	Positioning	Drift fault	Bosman et al. (2015) [82]
	NMF	Distributed	Application of NMF to fault diagnosis of soil moisture sensors	Humidity	Soft fault	Ludeña-Choez et al. (2014) [14]
	PCA	-	Introducing NN as classifiers into PCA fault diagnosis models	Temperature, air volume	Soft fault	Zhu et al. (2020) [85]
	Expert system	Distributed	Fuzzy rule fault node classification and management scheme to save energy	-	Hard fault	Yan et al. (2016) [90]
		-	The three-input FIS sensor hardware fault diagnosis model improves the accuracy	-	Hard fault	Li et al. (2017) [91]
	SVM	-	Developed a multi-SVM model for sensor fault diagnosis	Dissolved oxygen	Hard fault	Liao et al. (2017) [95]
		distributed	The OS-LSSVM sensor fault detection method addresses the lack of sparsity in SVM	Gyroscope	Bias fault	Li et al. (2017) [96]
			An LS-SVM model is proposed, which reduces the computational complexity	Temperature	Hard fault	Si et al. (2019) [97]
	ANNs	distributed	A BP model is built with recent local data, which can be used for online detection	Temperature	-	Yang et al. (2014) [103]
		-	Introducing wavelet denoising into BPNN to improve the fault diagnosis rate	Temperature	Hard fault	Deng et al. (2016) [104]
		-	fault diagnosis of HVAC sensors based on AANN	Water temperature	Drift and bias fault	Han et al. (2020) [105]
Deep Learning-based	AE	distributed	A three-layer network AE fault diagnosis system is designed, which can diagnose faults at the sensor	Temperature humidity	Spike and bias fault	Rumelhart et al. (2018) [110]
		-	A normalized sparse AE model is proposed to solve the variant feature problem of self-extracted features	-	-	Hu et al. (2018) [111]
		Centralized	A supervised SAE model is proposed to achieve higher accuracy at a lower number of iterations	Temperature	Soft fault	Shi et al. (2020) [112]
	DBN	distributed	A method based on DBN and generalized likelihood ratio test is proposed	Thermocouple	Soft fault	Lu et al. (2017) [118]
			The extended DBN model uses the strategy of repeatedly stacking the original data in the training phase to extract useful information fully	-	-	Luo et al. (2020) [119]
	RNN	distributed	RNN models nodes, the dynamics of nodes, and the coupling with other nodes with a low false positive rate	Temperature	Drift fault	Loy-Benitez et al. (2008) [123]
		-	LSTM and data fusion methods for multi-sensor fault diagnosis	Angle, speed	Stuck and biased fault	Hinton et al. (2019) [124]
		-	A fault diagnosis scheme based on RNN is proposed and combined with linear parameter changes to improve robustness	Angle	Soft fault	Roux et al. (2020) [125]
	CNN	distributed	The proposed CNN method enables fault diagnosis of multiple sensors with the same computational cost	-	Soft fault	Wang et al. (2020) [129]
		-	A CNN-RF method is proposed to improve the diagnostic success rate and reduce information loss in noisy environments	Hydrogen	Soft fault	Yang et al. (2020) [130]
		distributed	Multi-layer pooling classifiers replace the fully connected layers of CNN, reducing the parameters and the risk of overfitting	Acceleration	-	Hochreiter et al. (2022) [131]

Due to the fact that sensor data are collected at various time points with a high temporal correlation, the RNN model has been extensively studied in sensor fault diagnosis. Azzam et al. [132] utilized RNN to model sensor nodes, node dynamics, and sensor node coupling to achieve sensor node fault diagnosis. This method had a lower early false alarm rate than the Kalman filter. Lei et al. [133] adopted LSTM for wind turbine sensor fault diagnosis. The method can be extended to multi-sensor fault diagnosis through data fusion. In addition, the method was robust under limited data conditions, and its performance was superior to SVM and other approaches. Long et al. [134] proposed an RNN-based satellite sensor fault diagnosis scheme. They combined the linear parameter change method with the based model method to improve the robustness of fault diagnosis and the accuracy of the scheme. To solve the overfitting problem of deep neural networks in fault diagnosis, Xia et al. [135] implemented dropout in the LSTM model to prevent overfitting and enhance the training process’s efficacy.

Since deep learning automatically extracts features from the original data, there is no mapping between the extracted features and the fault mechanism. Although the deep learning model is suitable for resolving the big data characteristics of large-scale sensors, its training speed is slow and consumes computing resources. Therefore, the deep learning model is not typically executed directly on sensors but on edge nodes or the cloud.

#### 5.3.4. Convolutional Neural Networks (CNN)

CNNs are a significant subfield of deep learning. A CNN has an input layer, a convolution layer, a pooling layer, a full connection layer, and an output layer. It is capable of auto-extracting features and is both robust and generalizable. CNN is currently used primarily for feature extraction of two-dimensional and three-dimensional image sequences [136]. Still, because CNN has the characteristics of lattice and convolution operations and is suitable for multisensor data processing, some researchers have introduced CNN to the field of fault diagnosis [137]. Debasish et al. [138] proposed a CNN fault diagnosis method to diagnose multisensor faults at the same calculation cost to reduce model training time and cost. Sun et al. [139] proposed a CNN-RF sensor fault diagnosis method, which converted the original sensor signal into a grey matrix image, and the CNN is used to automatically extract the features of the gray matrix image, reducing the loss of effective information and improving the accuracy Muneer et al. [140,141] proposed an attention-based deep convolutional neural network (DCNN) architecture to predict the RUL of turbofan engines. They used multivariate time information to extract features, which significantly improved the prediction performance of the model. Zhang et al. [142] replaced the conventionally used fully connected layers in traditional CNN with a multilayer pool classifier, thereby reducing the number of network parameters and the risk of overfitting. The method performed a fault diagnosis on the acceleration sensor, with high fault-diagnosis accuracy and low computing resource consumption.

CNN’s ability to process two-dimensional and three-dimensional image data is its primary benefit. Muneer et al. [141] proposed a data-driven predictive model based on deep neural networks (DNNs), using sliding time window technology to prepare data, which does not require prior knowledge of prediction or signal processing. Currently, research in fault diagnosis focuses primarily on machinery, particularly rotating machinery, while research on sensor fault diagnosis is limited. Some researchers attempt to convert one-dimensional sensor data into a two-dimensional form, but this may destroy the spatial correlation of the original data and result in the loss of some error-related data. CNN is also suitable for processing large sample sizes but has a problem with overfitting small sample fault data.

The aforementioned methods for fault diagnosis are highly accurate, but each has advantages and disadvantages. The model-based method for fault diagnosis is highly interpretable. It does not require a large amount of data, but the model is complex and highly professional, which is unsuitable for daily agricultural applications. The fault diagnosis method based on artificial intelligence does not require an accurate mathematical model, and the accuracy of the diagnosis is high. However, the model has high requirements for calculation, storage, and other resources and requires a large amount of historical data. However, In daily agricultural activities, many quantity monitoring systems tend to collect and store real-time data, and insufficient amounts of historical data may reduce the accuracy of the model. The fault diagnosis method based on deep learning overcomes the flaw of the manual feature extraction method based on machine learning, and the model accuracy is higher; however, the method for fault diagnosis based on deep learning has flaws. To ensure the accuracy of the fault diagnosis model, it must be debugged and simulated multiple times, which wastes a significant amount of human resources and requires more resources such as computation and storage. Table 4 displays the advantages and disadvantages of each method.

### 5.4. Edge Computing-Based Fault Diagnosis Method

Edge computing is a new computing model that deploys computing and storage resources (such as Cloudlets, and fog nodes) to networks closer to mobile devices or sensors. IoT-based edge computing falls into three main categories: local devices adapted to specific definitions and good targets, local data centers providing significant data processing and storage capacity, and regional data centers closer to the data source. In response to the limitations of agricultural land, edge computing can be applied to work over long distances, and the processing of data generated by sensors through edge computing can improve response times, reduce the amount of communication transmitted to the cloud, and avoid latency problems in data processing [143].

Control monitoring of data using edge computing allows data consistency and integrity analysis, identification, and removal of erroneous data. When an edge device fails, the edge computing system can notify the user which component is at fault, which facilitates sensor fault detection. Sun et al. proposed a real-time detection algorithm based on edge computing and cloud computing to improve the average repair time by pre-processing the original video data when performing fault diagnosis on video surveillance systems [144]. Li et al. point out that sensor-based data collection systems suffer from significant latency and data redundancy, and edge computing is often applied closer to the source of data generation, i.e., data processing is performed locally, sharing the reduced data redundancy and solving latency problems [145]. Since edge computing will control in real-time, monitor the process of data processing, and send the analysis results to the cloud, it is convenient for managers to view the working status of sensors in time and make judgments on whether the sensors produce faults. To solve some of the problems of wireless sensor data collection, wireless sensors are combined with edge computing to establish data collection algorithms. In wireless sensor networks, through edge servers, invalid data is filtered, sensing parameters are obtained, and the collected data is transmitted to the cloud or edge servers to improve sensor networks’ intelligence and computing capability [146], laying the foundation for its fault diagnosis. Akhtar et al. proposed that the data distribution of edge computing was heterogeneous and required distributed data processing and storage. On the basis of edge computing, they proposed High-Performance Computing (HPC), which can identify data anomalies by performing quality checks at the edge layer, which can help troubleshoot sensors to ensure accuracy and effectiveness. Users can view, analyze, and operate the data stored in the cloud and use the machine learning tools stored in the cloud database to analyze the data and make judgments when the sensor is faulty [147].

## 6. Challenges and Future Development

Smart agriculture with Agriculture 4.0 at its core has become the current focus of agricultural development on a global scale. With the development of IoT, big data, wireless sensing, and other technologies, intelligent equipment in agriculture continue to increase, the types of faults are becoming more complex and diverse, and the demand for Ag-IoT-based fault diagnosis technology is rising. Some fault detection methods based on a single target can no longer meet the demand. First, it is necessary to consider the economic benefits and feasibility of the diagnostic system for farmers and try to ensure the system’s simplicity while ensuring that the diagnosis is correct. At the same time, the amount of information in the agricultural field is huge, which is a great test for the stability of any system. The main goal of future fault diagnosis technology is to achieve early detection and treatment of sensor faults while ensuring system stability. Therefore, we provide a summary of the fault diagnosis areas that will receive future attention.

### 6.1. Edge Computing

Intelligent fault diagnosis requires high real-time performance and hardware resources. Practical issues such as network unavailability, full bandwidth, or insufficient sensor computing and storage resources are frequently encountered in agricultural scenarios, making it difficult for centralized and distributed fault diagnosis strategies to meet sensor fault diagnosis requirements. Edge computing is a remedy for the aforementioned issues. Edge computing allows faster response times and more secure data transmission and processing than other sensor fault diagnosis methods. At the same time, edge computing can be combined with deep learning algorithms to reduce model training time and make sensor fault diagnosis more efficient [148]. Edge computing transfers a portion of the central server’s computing and storage resources to edge nodes and collects sensor data on the edge computing platform to develop fault diagnosis models. Unmanned plant protection machines and inspection robots are two examples of agricultural equipment that transports substantial resources. Consequently, using such equipment as a platform for edge computing does not increase costs and satisfies the requirements for fault diagnosis.

### 6.2. Satellite Communication

Few base stations and low network coverage are characteristics of Ag-IoT, which prevents the wireless sensor node from sending data to the central node, using the centralized fault diagnosis strategy, and sending fault information to the central node using the distributed fault diagnosis strategy. In addition, agricultural sensors are frequently deployed in harsh environments, and environmental factors such as sudden changes can cause network disruptions and other problems. With the proposal of Starlink, people began to investigate the commercial application of satellite Internet and the use of satellite communication to solve the problem of no network in sensor deployment areas [149]. Satellite communication has the advantages of long communication distance, being unrestricted by natural conditions, and having high stability, which guarantees the network’s continuous availability in sensor fault diagnosis.

### 6.3. Hybrid Fault Diagnosis Model

The sensor system in Ag-IoT is frequently dynamic, uncertain, open, vulnerable to attack, and concurrently susceptible to multiple faults. If only a single fault diagnosis method is used, issues such as low accuracy, poor generalization ability, and incomplete diagnosis arise, making it difficult to achieve accurate diagnosis results. The method for fault diagnosis based on the fusion of multiple technologies can be studied. Combining a model-based approach and deep learning can enhance a diagnostic system’s robustness, accuracy, and dependability while decreasing uncertainty. This method can not only expand the fault ability based on deep learning but also fully utilize the interpretability advantage of the model-based method, which aids technicians in understanding the fault type and origin.

## 7. Conclusions

This paper reviews fault diagnosis technology in the context of artificial intelligence technology and big data. The concept of sensors and sensor networks in Ag-IoT are introduced, and common sensor fault types and fault diagnosis strategies in Ag-IoT are listed. In addition, the research and application status of three sensor fault diagnosis technologies are summarized: model-based, artificial intelligence-based, and deep learning-based. The methods are compared and their advantages and disadvantages are discussed. Through the analysis of this review, the following conclusions are drawn, and suggestions for future work are put forward.

(1) Presently, sensors are usually deployed in harsh environments, which are extremely vulnerable to environmental impact, resulting in inaccurate data collection and irreversible impact on sensors. In the future, sensors will be further developed, less affected by the environment, and can use limited resources to work for a long time; The output format codes between different sensors have developed from the original disunity to the standardized format.

(2) Deep learning has unique advantages, although the research in the field of sensor fault diagnosis is still in its infancy, it has a good development prospect. It is necessary to improve the system’s robustness to meet the changes in the conditions in practical applications.

(3) The commonly used single-fault diagnosis methods often encounter many problems: resource limitation problems, data transmission problems, node optimization problems, data security problems, and so on. In the future, fault diagnosis methods based on the integration of multiple technologies can be studied, such as adding edge computing and satellite communication and combining expert systems and deep learning to shorten the training time by using known rules through transfer learning, which could improve the accuracy of sensor fault diagnosis and reduce the frequency of faults to ensure the stability and reliability of intelligent agricultural measurement and control systems.

## Figures and Tables

**Table 1:** Comparison of data transmission technologies commonly used in agricultural IoT.

	Transmission Distance	Power Consumption	Delay Time	Advantage	Disadvantage
Bluetooth [32]	<10 m	Low	<1 s	Low cost	Incompatible protocols between different devices
Wi-Fi [33]	<50 m	High	<1 s	Easy fault location	The transmission process is unstable
ZigBee [34]	10–100 m	Low	<1 s	High security	High cost
LoRa [35]	<10 km	Low	1 s	The networking mode is flexible and can connect multiple nodes	Users need to form their own network
NB-IoT [36]	<25 km	Low	6–10 s	Wide coverage	Low data transfer

**Table 2:** Representative agricultural sensors in a variety of Ag-IoT applications.

Classification	Metrical Information	Principle of Measurement	Typical Product	Related References
Environmental information	Ammonia gas	Electrochemistry	Winsen, ME3-NH3	Smith et al. (2020) [41]
	Carbon dioxide	Electrochemistry, optics	Hanwei, MH-Z19	Chen et al. (2012) [42]
	Oxygen	Electrochemistry	Renke, RS-O2	Levintal et al. (2022) [43]
	Air temperature	Pyroelectricity	METER, ECT	Fisher et al. (2010) [44]
	Air humidity	Electrochemistry, electromagnetism	Renke, RS-WS-N01-2	Yang et al. (2013) [45]
	Soil temperature	Pyroelectricity	METER, RT-1	Zhang et al. (2011) [46]
	Soil humidity	Electronics and electromagnetics	METER, ECH2O EC-5	Antonacci et al. (2018) [47]
	PH value in the water body	Electrochemistry	Renke, RS-PH-N01-A-201	Akhter et al. (2021) [48]
	Intensity of illumination	Optics	METER, PYR	Hu et al. (2013) [49]
	Rainfall	Electronics and electromagnetics	METER, ECRN-100	Katya et al. (2020) [50]
	Poultry dust	Laser light scattering	Renke, RS-PM	Jae et al. (2008) [51]
Crop life information	Leaf humidity	Electromagnetism	METER, PHYTOS 31	Kamlesh et al. (2022) [52]
	Leaf temperature	Thermoelectricity	Bio Instruments, LT-1P	Ge et al. (2014) [53]
	Stomatal conductivity	Electronics, mechanics, optics	METER, SC-1	R. Valdés et al. (2015) [54]
	Electric conductivity	Electrochemistry	METER, HYDROS 21	Serrano et al. (2019) [55]
	Stem flow	Energy balance	Ecotek, SGDC	Singh N et al. (2020) [56]
	Canopy reflectance index	Optics	Ecotek, SRS-PRI	Giménez et al. (2021) [57]
	Stem growth	Mechanics	AWL, SD-5z	Ohana et al. (2022) [58]
Animal information	Body temperature	Optics/thermoelectrics	Wuhe, W630	Wi et al. (2019) [59]
	Amount of exercise	Electromagnetism	Quantified Ag	Lawson et al. (2022) [60]
	Locator	GPS/BD/GLONASS	Naviecare, GA5201	Buerkert et al. (2009) [61]
	Body weight	Electronics and electromagnetics	OMEGA, TQ101	He et al. (2023) [62]

**Table 4:** Comparison of advantages and disadvantages of intelligent fault diagnosis methods.

		Advantages	Disadvantages
Model-based		Strong interpretability Good real-time performance	Poor universality Struggles to establish accurate models The amount of data processed is limited
AI-based	Statistical analysis [68]	Good interpretability No need to understand the structure and principle of the sensor system	Poor real-time performance It is easy to misjudge when the data fluctuates greatly Suitable only for working with univariate data
	Expert system [75]	Strong fault tolerance Autonomous learning ability Good performance in nonlinear systems	Lack of versatility and flexibility Systems are independent of each other, making it difficult to handle multivariate data
	SVM [81]	Strong ability to handle small samples and nonlinear data	Weak multi-classification ability Poor real-time performance
	NN [83]	Good universality High diagnostic accuracy Be able to handle multivariate data	High requirements for data volume
DL-based	AE [95]	Good robustness Low demand for training data samples	Extract irrelevant features to increase the amount of computation Poor real-time performance
	DBN [103]	Strong ability to process high dimensional and nonlinear data Automatically acquire advanced features and reduce training time	Loss of useful information in feature extraction Poor performance in terms of accuracy
	RNN [107]	Strong ability to process time series data Be able to handle large amounts of data	The model is prone to gradient disappearance High demand for training data Low training speed
	CNN [115]	Be able to handle large volumes of multivariate data Training time and cost are low	Overfitting occurs when processing small sample data

## Data Availability

Not applicable.
